# Supplementary material for: Protocol for a scoping review on technology use and sexual and gender minority youth and mental health
Source: PLoS One. 2024 Jan 26;19(1):e0291539. doi: 10.1371/journal.pone.0291539 (PMC10817184; doi:10.1371/journal.pone.0291539)
Supplement: S1 Appendix — (DOCX) [file pone.0291539.s001.docx]

Appendix 1: Search Strategy for PsycINFO

| # | Searches |
| --- | --- |
| 1 | [LGBT* or GLBT* or LGB or LGBs or LGBQ* or M2F or GLB or GLBQ* or GLBs or heteroflexible* or GBMSM* or msm or men who have sex with men or TGNC* or YTW or Gay or gays or lesbi* or bisexual* or transgender* or bicurious* or transpeople* or asexual* or women loving women or women who have sex with women or WLW or transvestit*e or cross sex* or crosssex* or crossgender* or F2M or transperson* or transsexual* or homosexual* or intersex* or queer* or androgyn* or genderqueer or two spirit or pansexual* or demisexual* or asexual* or SGM or SOGI or nonbinary or non-binary or 2SLGBT* or aromantic* or crossdresser* or demisexual* or FTM or MTF or genderfluid or omigender* or pangender* or same gender loving or third gender or WSW or [[gender* or sex or sexual] adj2 [change or dysphoria or reversal or identit* or reassign or transform* or transition* or minorit* or binary]]].tw. |
| 2 | sexual minority groups/ or exp gender identity/ or androgyny/ or gender dysphoria/ or sexual orientation/ or asexuality/ |
| 3 | 1 or 2 |
| 4 | [youth* or teen* or adolescen* or puberty or pubescen* or emerging adult* or [young adj2 [people or individual or individuals or person or persons or man or men or male or female or woman or women or population* or adult*]] or [[high school or highschool or secondary school or middle school or junior high or jr high or college or university* or post secondary or postsecondary] adj2 student*]].tw. |
| 5 | exp college students/ or high school students/ or middle school students/ |
| 6 | 4 or 5 |
| 7 | 3 and 6 |
| 8 | ["sexual and gender minority youth" or sgmy].tw. |
| 9 | 7 or 8 |
| 10 | [information communication technology* or ICT or ICTs or information system* or internet or digital* or virtual* or web or cyber* or online or technolog* or digital media or social media or electronic communication or computer mediated communication or telecommunication* or podcast* or twitter or facebook or youtube or discord or tumblr or twitch or reddit or messenger or instagram or 4chan or tik tok or snapchat or wechat or qq or douyin or weibo or telegram or pinterest or qzone or kuaishou or quora or phone* or mobile app* or telephone* or smartphone* or cellphone* or mobile* or chat or blog* or video* or email* or texting or sms or text messaging or television* or tv or radio*].tw. |
| 11 | technology/ or "information and communication technology"/ or digital technology/ or exp electronic communication/ or exp mobile technology/ or exp internet/ or communications media/ or exp digital media/ or exp telecommunications media/ or online community/ or exp human technology interaction/ |
| 12 | 10 or 11 |
| 13 | [mental health or mental illness* or mental disorder* or mental hygiene or psychological or psychiatric disorder* or psychiatric illness* psychoses or psychosis or well-being or wellbeing or safe* or identity formation or identity development or depress* or anxiety or stress or stressor* or traum* or post-traumatic or posttraumatic or ptsd or grief or bereavement or distress or burnout or despair or fear or panic or worry or isolation or loneliness or [[alcohol or substance* or drug* or marijuana] adj2 [abuse or "use" or usage or using]] or drinking or phobia* or cyberchondria or hypochondriasis or coronaphobia or mania* or trichtillomania or obsession or obsessive compulsive disorder or ocd or self harm or self injury or suicid* or violence or cope or coping or resilience or resilient or social support].tw. |
| 14 | exp mental health/ or exp mental disorders/ or exp bereavement/ or exp coping behavior/ or panic/ or "Resilience [Psychological]"/ or exp well being/ or social isolation/ or exp suicide/ or exp anxiety/ or exp self-injurious behavior/ or distress/ or psychological stress/ or posttraumatic stress/ or emotional trauma/ or traumatic reactions/ or posttraumatic growth/ or exp self-concept/ or identity formation/ |
| 15 | 13 or 14 |
| 16 | 9 and 12 and 15 |
